# Utilizing Machine Learning for Diagnostic Assistance of Pediatric Sepsis and Septic Shock in Resource-Limited Settings

**DOI:** 10.3390/pediatric18040088

**Published:** 2026-07-03

**Authors:** Kaden Bunch, Shamsun Nahar Shaima, Gazi Md. Salahuddin Mamun, Sai Gopal Jarabana, Monique Gainey, Abu Sayem Mirza Md. Hasibur Rahman, Alicia Genisca, Atin Jindal, Nidhi Kadakia, Monira Sarmin, Farzana Afroze, Adam C. Levine, Mohammod Jobayer Chisti, Stephanie Chow Garbern

**Affiliations:** 1 Warren Alpert Medical School of Brown University, Providence, RI 02903, USA; adam_levine@brown.edu; 2 International Centre for Diarrhoeal Disease Research, Bangladesh (icddr,b), Dhaka 1000, Bangladeshgazi.mamun@icddrb.org (G.M.S.M.);; 3 Department of Emergency Medicine, Warren Alpert Medical School of Brown University, Providence, RI 02903, USAstephanie_garbern@brown.edu (S.C.G.); 4 Virginia Tech Carilion School of Medicine, Roanoke, VA 24016, USA; 5 Division of Hospital Medicine, Brown University Health System, Providence, RI 02903, USA; 6 Department of Emergency Medicine, Yale School of Medicine, New Haven, CT 06510, USA

**Keywords:** pediatric sepsis, machine learning, low- and middle-income countries, diagnostic classification, phoenix sepsis score, clinical decision support, Bangladesh

## Abstract

Background: Sepsis is a leading cause of pediatric mortality worldwide, disproportionately affecting children in low- and middle-income countries (LMICs). However, timely recognition of potential sepsis and access to healthcare resources needed to diagnose pediatric sepsis according to international guidelines are challenging in LMICs. This exploratory study aimed to develop machine learning (ML) models to detect pediatric sepsis and septic shock using a simplified set of clinical data contextualized for practical use in resource-limited settings. Methods: This was a secondary analysis of an observational study of 100 children with potential sepsis admitted to a non-profit referral hospital in Dhaka, Bangladesh. The outcomes were sepsis as defined by a Phoenix Sepsis Score (PSS) ≥ 2 and septic shock (sepsis plus PSS cardiovascular sub-score ≥ 1). Models were trained using either clinical + laboratory variables or clinical-only variables. A single 24 h worst-value assessment window was derived per patient; stratified 5-fold cross-validation was used to maintain class proportions across the training and test folds. Model performance was assessed using area under the precision–recall curve (AUPRC) and area under the receiver operating characteristic curve (AUROC) with 95% confidence intervals (CIs) derived from a 2000-resample patient-level bootstrap of out-of-fold classifications. Logistic regression coefficients were used to assess feature contributions. Results: For sepsis classification, the non-laboratory model achieved an AUPRC of 0.942 (95% CI: 0.884–0.979) and an AUROC of 0.945 (95% CI: 0.890–0.983), with comparable performance from the clinical + laboratory model (AUPRC 0.941, 95% CI: 0.880–0.981; AUROC 0.945, 95% CI: 0.881–0.986). For septic shock, AUROCs of 0.870 (95% CI: 0.761–0.952) and 0.878 (95% CI: 0.758–0.967) were observed. However, these estimates should be interpreted cautiously, given the low prevalence (23%) and absence of external validation. SpO_2_:FiO_2_ ratio, GCS, and systolic blood pressure were consistently strong predictors across models. Conclusions: ML models using pragmatic clinical variables demonstrate preliminary diagnostic performance, with the non-laboratory model showing discrimination comparable to models incorporating laboratory data. Logistic regression demonstrated the most stable performance and may represent an early proof of concept for assistive diagnostic support. However, these models are not clinically usable without external validation. These findings are hypothesis-generating; external validation in larger, independent cohorts is essential before any clinical use, particularly for septic shock.

## 1. Introduction

Sepsis continues to be a leading cause of child mortality worldwide, with wide variations in mortality rates ranging from <5% to over 40% depending on contextual factors such as epidemiology, health care access and availability [1,2,3]. Pediatric sepsis deaths disproportionately occur in low- and middle-income countries (LMICs) for a multitude of reasons, including limited access to diagnostic and management resources, contributing to delayed recognition of sepsis among children with infections [4]. Early recognition of sepsis, assessment of the severity of organ dysfunction, and timely evidence-based intervention with antibiotics, judicious fluid resuscitation, and respiratory and cardiovascular support are crucial steps in sepsis care, which have been shown to reduce sepsis mortality rates [5].

The importance of early recognition and prognostication in pediatric sepsis care is reflected in the development of numerous scoring systems, such as Systemic Inflammatory Response Syndrome (SIRS), Sequential Organ Failure Assessment (SOFA), Quick SOFA (qSOFA), Pediatric Risk of Mortality Score III (PRISM-3), Pediatric Logistic Organ Dysfunction II (PELOD-2), and the Pediatric Index of Mortality III (PIM-3), which have been used to identify at-risk patients, as well as to predict adverse outcomes and mortality risks [6,7,8]. Additionally, the definition of sepsis has evolved over time, moving from SIRS-based criteria to organ dysfunction-based criteria. The International Consensus Criteria for Pediatric Sepsis and Septic Shock was updated in 2024, which defines sepsis based on the newly developed Phoenix Sepsis Score (PSS), which comprises four organ system components: respiratory, cardiovascular, coagulation, and neurologic, to classify sepsis (PSS ≥ 2) and septic shock (sepsis with ≥1 cardiovascular point) [6]. The PSS is highly promising, achieving an area under the receiver operating characteristic curve (AUROC) of 0.71–0.92 in the original multi-country validation sets (including both high- and low-resource settings) for predicting mortality [9]. The PSS also offers advantages over scores such as PELOD-2 and pSOFA by reducing the requirements for laboratory and clinical inputs, making the score more feasible for use in lower-resource settings, although implementation of PSS-based sepsis protocols into routine clinical use in LMICs is still in the early stages.

Despite these recent advances in sepsis diagnosis, scores such as the PSS remain challenging in actual practice in many settings, due to the constraints on resources needed to calculate all components of the score, as well as on health personnel who often have many competing demands on their time. Components of the PSS include platelet count, international normalized ratios (INR), D-dimer, fibrinogen, and lactate, some or all of which may be impractical or even impossible to obtain in lower-resourced clinical settings, particularly in LMICs [10,11]. There is an urgent need for more feasible and simplified models for these settings. While the authors of the PSS have tried to ensure redundancy in the score to account for the potential lack of coagulation parameters, clinical heterogeneity, and varying resource availability in hospitals (the ability to perform some laboratory diagnostics may even vary on a day-to-day basis), this can further complicate the external validity of the PSS and face many of the same barriers to implementation as older scoring systems [12].

In recent years, machine learning (ML) methods have been increasingly used to help improve the clinical prediction and classification of a wide variety of medical conditions [13,14]. Studies within adult populations in high-resource settings using ML for sepsis have shown particularly promising results, identifying complex patterns that may not be readily apparent compared to more traditional methods [15]. However, despite significant advancements in ML applications in healthcare, the use of ML models remains under-validated in LMICs, particularly in pediatric populations, due to inequities in health data availability, variable ability to assess or document clinical and laboratory parameters, and limited infrastructure to implement complex ML models effectively [13].

The aim of this exploratory study was to develop ML models for the diagnosis of sepsis and septic shock (as defined using PSS) within a pediatric population, using only a limited set of features that could reasonably be collected in lower-resourced clinical settings in LMICs. Models such as these could be used as an operational approximation of PSS or other more complex scores and be implemented through mobile health (mHealth) platforms, leading to more widespread uptake and greater recognition of pediatric sepsis in LMICs.

## 2. Materials and Methods

### 2.1. Study Design and Patient Population

This was a secondary analysis of a prospective observational study of children with potential sepsis admitted to the intensive care unit (ICU) of the International Centre for Diarrhoeal Disease Research, Bangladesh (icddr,b) in Dhaka, Bangladesh, from February to December 2022. Eligible patients ranged from 2 months (prematurity-corrected) to 17 years of age and satisfied the criteria for potential sepsis, defined as physician suspicion of infection coupled with at least two SIRS criteria (one of which had to be either an abnormal temperature or leukocyte count), based on the international sepsis definition guideline and hospital protocol at the time of the study [16]. The SIRS criteria were defined as follows:

1. Temperature >38.5 °C or <36 °C;
2. Heart rate above two standard deviations adjusted for age range or below the 10th percentile in children under one year;
3. Respiratory rate more than two age-adjusted standard deviations above normal, or mechanical ventilation required for an acute process;
4. Elevated or depressed leukocyte count adjusted for age or >10% immature (band) forms [16].

A full description of the study cohort has been previously published [17].

### 2.2. Ethical Approval

Ethical approval was obtained from the icddr,b Institutional Review Board (comprising the Research Review Committee and Ethical Review Committee) and the Rhode Island Hospital (RIH) Institutional Review Board (IRB). Study procedures were conducted in accordance with the Declaration of Helsinki. The parent/guardian of each eligible patient was approached for participation, and written informed consent and assent for patients aged 11–17 years were obtained in the local language (Bangla). As this study constitutes a secondary analysis of a previously approved dataset, no additional consent was required beyond that obtained in the parent study [17].

### 2.3. Study Outcomes

The co-primary outcomes were sepsis and septic shock, with each treated as a binary (dichotomous) outcome derived from the 2024 Phoenix Sepsis Score (PSS) [6]. Sepsis was defined as a PSS total score ≥ 2, encompassing four organ system sub-scores: respiratory, cardiovascular, coagulation, and neurological. Septic shock was defined as meeting sepsis criteria (PSS ≥ 2), with an additional cardiovascular sub-score ≥ 1 reflecting the presence of cardiovascular organ dysfunction. Outcome labels were assigned based on the most abnormal values recorded within the 24 h assessment window. The PSS was calculated independently of model classification and was not used as a model input.

### 2.4. Data Collection

Trained study nurses and physicians collected demographic and clinical data such as age, sex, weight, and comorbidities and performed laboratory testing, including complete blood count (CBC), coagulation panel (PT/INR), arterial (or venous if arterial was unable to be collected) blood gas, electrolytes, glucose, lactate, and renal and hepatic function panels. Laboratory tests were conducted on the day of admission and then twice daily throughout the patient’s stay. Clinical assessments, including the Glasgow Coma Scale (GCS) and manual vital signs (BP, HR, RR, Temp, and SpO_2_) measured by an experienced nurse, medication administration (including vasopressors), general appearance, and the use of respiratory support (including mechanical ventilation), were recorded every four hours. SpO_2_ was measured with a portable pulse oximeter (Masimo RadG^®^), and temperature was measured using a digital thermometer (rectal for children <2 years, or axillary if >2 years). D-dimer and fibrinogen were not available and, therefore, were scored as 0 according to the PSS’ guidelines for use [6].

Patient data was compiled, matched to unique identifiers, and organized into a single 24 h assessment window per patient, anchored to each patient’s recorded admission timestamp. Within this window, measurements were first aggregated into 4 h time slots. For each 24 h window, the worst (most physiologically abnormal) value across the slots was retained to capture peak derangement, consistent with prior approaches [18,19]. Categorical or treatment variables were summarized as the highest recorded level or the presence of the intervention. Missing data were handled sequentially, consistent with a missing-at-random (MAR) assumption: (1) laboratory values timestamped before the formal study enrollment (e.g., admission workup specimens drawn before nurse assessment, such as the complete blood count) were attributed to the 24 h assessment window to ensure no admission-workup data was excluded; (2) within-patient linear interpolation was applied across time slots for continuous variables, with a maximum allowable gap of 48 h, using only each patient’s own longitudinal data; (3) remaining gaps were filled by last-observation-carried-forward and next-observation-carried-backward (LOCF/NOCB); and (4) Phoenix score components with no available measurement were scored as 0 points, per published PSS guidelines for unavailable data [6]. Feature standardization (zero-mean, unit-variance scaling) was performed strictly within each cross-validation training fold to prevent information leakage.

### 2.5. Feature Selection

Two distinct feature sets were created for comparative analysis following data cleaning and imputation. Feature sets 1 and 2 were developed using variables reasonably feasible for measurement in resource-limited hospital settings, as deemed by consensus among the study co-authors based in LMICs or with extensive experience working in LMICs (Table 1). Feature set 1 included both clinical and laboratory data (applicable to settings with access to some laboratory diagnostics), while feature set 2 used only clinical variables from feature set 1, excluding laboratory data altogether (applicable to the lowest-resourced settings without reliable laboratory access).

The models were trained using the following feature sets:

- Feature Set 1 (Clinical and Laboratory Variables): SpO_2_:FiO_2_, platelet count, respiratory rate, systolic blood pressure, white blood cell count, Glasgow Coma Scale, heart rate, temperature, hemoglobin, age;
- Feature Set 2 (Non-Laboratory Variables Only): SpO_2_:FiO_2_, respiratory rate, systolic blood pressure, Glasgow Coma Scale, heart rate, temperature, age.

### 2.6. Model Training and Evaluation

Seven ML models—Decision Tree, Random Forest, Support Vector Machine (SVM), Kernel SVM, Naïve Bayes, K-Nearest Neighbors (KNN), and Logistic Regression—were assessed for their capacity to classify sepsis and septic shock as defined by the 2024 Phoenix criteria. For each feature set, classifications were made separately for (1) sepsis and (2) septic shock.

Given the relatively small sample size (N = 100; 40% meeting PSS sepsis criteria, 23% meeting PSS septic shock criteria within the 24-h assessment window), a repeated, stratified, 5-fold cross-validation scheme was implemented using the RepeatedStratifiedKFold strategy from scikit-learn (v0.24.2) [20]. This ensured that each patient’s single observation was assigned entirely to either the training or test fold, while preserving class proportions. Each model was optimized within this cross-validation framework using Optuna (v4.9.0), a Bayesian hyperparameter optimization library, to select model-specific parameters (e.g., number of trees, depth, and regularization parameters) [21]. Optimized hyperparameters for each model are provided in Table S1.

Before evaluating the model, all features were standardized using scikit-learn’s StandardScaler prior to model fitting [20]. Three classification thresholds were pre-specified: (1) An initial fixed threshold of 0.50 was used as the primary reference for the multi-model comparison table. A threshold of 0.50 was selected, as it represents a transparent, leakage-free, pre-specified operating point under class-weighted training and is appropriate for comparing models on equal footing, though it does not imply this would be the deployed clinical threshold. Following identification of a consistent model, 2 further thresholds were specified: (2) An inner cross-validation F1-optimal threshold, selected by maximizing F1 on out-of-fold classifications within each training fold only (never on the held-out test fold), to demonstrate performance under optimized threshold conditions without data leakage, and (3) a screening threshold of 0.30, reflecting the high-sensitivity prioritization appropriate for a diagnostic screening tool. Confusion matrices with sensitivity, specificity, positive predictive value (PPV), and negative predictive value (NPV) at all three thresholds for the headline model were then recorded.

Feature contributions were examined using correlation matrices, pairwise plots, and model-specific feature importances or coefficients. For models with interpretable coefficients (e.g., logistic regression), standardized coefficients were used to quantify the effect of a one-standard-deviation increase in each predictor on the log-odds of sepsis or septic shock [22].

Septic shock was a rare outcome in our cohort. As such, AUPRC was considered the primary performance metric, as it provides a more sensitive measure of model performance for rare outcomes compared to AUROC [23]. Python (v3.10) was used for all analyses, and figures and tables were generated using Matplotlib (v3.11.0) and Seaborn (v0.13.2). Threshold-optimized classification, cross-validated metrics, and hyperparameter details are included in the Supplementary Materials to facilitate reproducibility.

All code used for data processing, model training, and evaluation is available in the GitHub Repository (https://github.com/kadenbunch/pediatric-sepsis-ml, accessed on 27 July 2026) [24].

## 3. Results

A total of 100 patients were enrolled in the parent study cohort (Table 2). The median age was 8 months [IQR 5–18], and 59% were male. The in-hospital mortality rate was 24%, and 40% met the criteria for sepsis (PSS ≥ 2) over the initial 24 h, with 23% developing septic shock over the hospital course. Most patients (84%) had no reported past medical comorbidities.

### Model Performance

Among the models, Logistic regression consistently demonstrated strong performance across feature sets and was, therefore, selected for primary interpretation and feature effect analysis (Table 3). Performance metrics for all evaluated models and feature sets are summarized in Table S2.

**Figure 1:**
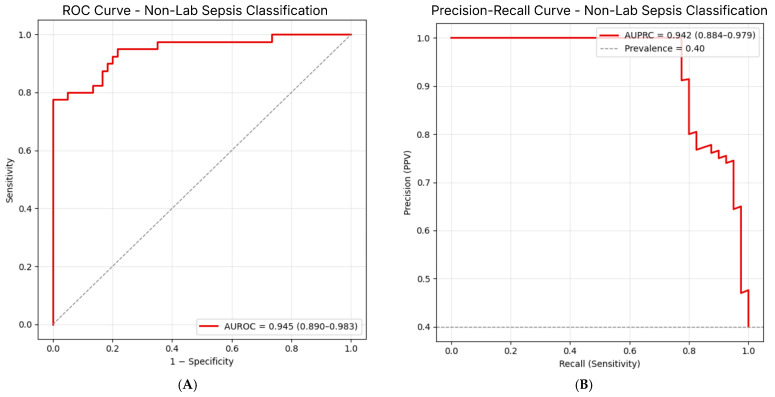
Receiver operating characteristic (ROC) curve (**A**) and precision − recall (PR) curve (**B**) for the logistic regression model classification of sepsis using the non-lab feature set. Curves were generated using aggregated out-of-fold predicted probabilities obtained from repeated stratified 5-fold cross-validation.

For sepsis classification, the clinical + laboratory feature set achieved an AUROC of 0.945 (95% CI 0.881–0.986) and an AUPRC of 0.941 (95% CI 0.880–0.981) (Figure 1). The non-laboratory feature set showed equivalent discrimination (AUROC 0.945, 95% CI 0.890–0.983; AUPRC 0.942, 95% CI 0.884–0.979). Full metrics for the LR models are presented in Table 3. ROC and precision–recall curves for the non-laboratory sepsis model are shown in Figure 1; curves for all feature sets are in Figure S1.

For septic shock classification, slightly lower overall metrics were observed compared to the sepsis models. However, these must be interpreted cautiously given the low prevalence (23%), small absolute number of events, and absence of external validation. For the clinical + laboratory feature set, logistic regression achieved an AUROC of 0.870 (95% CI 0.761–0.952) and an AUPRC of 0.793 (95% CI 0.621–0.908). The non-laboratory feature set showed similar performance (AUROC 0.878, 95% CI 0.758–0.967; AUPRC 0.822, 95% CI 0.664–0.929). The apparent equivalence of the non-laboratory model is likely a reflection of small sample size and outcome rarity rather than a genuine informational advantage; external validation is required to confirm these findings.

Calibration, assessed by the Brier score, ranged from 0.086 (95% CI 0.049–0.132) for the clinical + laboratory sepsis model to 0.163 (95% CI 0.149–0.178) for the non-laboratory sepsis model and from 0.141 (95% CI 0.119–0.166) to 0.119 (95% CI 0.080–0.171) for the septic shock models.

An exploration of threshold-dependent performance metrics for sepsis and septic shock classification across feature sets is presented in Table 4. At the default threshold of 0.50, the clinical + lab and non-lab models achieved identical sensitivity (0.825), with clinical + lab showing higher specificity (0.900 vs. 0.833). The F1-optimal threshold had no practical effect on the clinical + lab classification (threshold 0.453 produced identical TP/FP/FN/TN to 0.50), while the non-lab optimal threshold (0.522) improved specificity to 0.933 with a modest sensitivity reduction to 0.800. At the 0.30 screening threshold, clinical + lab retained a clinically favorable balance (sensitivity 0.900, specificity 0.850), whereas the non-lab model captured all positive cases (sensitivity 1.000) at the near-complete loss of specificity (0.083).

For septic shock, both models at 0.50 demonstrated equivalent specificity (0.870) with sensitivity of 0.739 (clinical + lab) and 0.783 (non-lab). F1-optimal thresholds were notably higher for septic shock than for sepsis (0.557 and 0.776, respectively), yielding improved specificity (0.922 and 0.948) at the cost of lower sensitivity (0.696 and 0.609). At the 0.30 screening threshold, clinical + lab achieved near-complete sensitivity (0.957, one missed case) with moderate specificity (0.468), while non-lab maintained a more favorable balance (sensitivity 0.826, specificity 0.701).

Table 5 presents standardized logistic regression coefficients and odds ratios for sepsis and septic shock classification across the clinical + laboratory and non-laboratory feature sets. For sepsis classification, the strongest associations in the clinical + laboratory model were observed for the SpO_2_:FiO_2_ ratio (β = −2.783, 95% CI −4.270 to −1.297; OR 0.062, 95% CI 0.014–0.273) and Glasgow Coma Scale (β = −1.596, 95% CI −2.874 to −0.318; OR 0.203, 95% CI 0.056–0.728). Systolic blood pressure showed a negative association of comparable magnitude (β = −1.561, OR 0.210, 95% CI 0.043–1.035). Among laboratory variables, platelet count (β = −0.984, OR 0.374, 95% CI 0.132–1.059) and WBC count (β = −0.639, OR 0.528, 95% CI 0.205–1.358) demonstrated negative associations, with the confidence intervals marginally including zero. In the non-laboratory sepsis model, SpO_2_:FiO_2_ ratio (β = −0.326, OR 0.722, 95% CI 0.426–1.221), GCS (β = −0.250, OR 0.779, 95% CI 0.451–1.346), and systolic blood pressure (β = −0.191, OR 0.826, 95% CI 0.505–1.352) retained the largest negative coefficient magnitudes; th remaining predictors in both sepsis models showed near-null effect estimates with wide confidence intervals.

For septic shock classification, in the clinical + laboratory septic shock model, negative associations were observed for systolic blood pressure (β = −0.512, OR 0.599, 95% CI 0.333–1.077), SpO_2_:FiO_2_ ratio (β = −0.395, OR 0.674, 95% CI 0.384–1.183), and GCS (β = −0.234, OR 0.792, 95% CI 0.438–1.430). The laboratory parameters in this model demonstrated near-null effects (hemoglobin OR 0.987, platelet count OR 0.849, WBC count OR 0.914). In the non-laboratory model, key associations included lower SpO_2_:FiO_2_ ratio (β = −1.458, 95% CI −2.357 to −0.558; OR 0.233, 95% CI 0.095–0.572) and lower systolic blood pressure (β = −2.513, 95% CI −4.304 to −0.722; OR 0.081, 95% CI 0.014–0.486).

Across outcomes, coefficient directionality was preserved between feature sets, with an attenuation of effect sizes observed in the clinical + laboratory sepsis model relative to the septic shock models. Standardized coefficient magnitudes are shown in Figure S2 to facilitate comparison of predictor importance across models, with full logistic regression equations for each outcome and feature set in Table S3.

## 4. Discussion

Despite recent improvements in pediatric sepsis detection globally, early identification of pediatric sepsis and septic shock using international criteria such as the PSS remains a major challenge in resource-limited settings due to the relative complexity of calculation. Early identification of potential sepsis, especially in lower-resourced health facilities where children often initially present, is critical to appropriately implement life-saving interventions or refer children to a higher level of care. Our findings demonstrate that ML models trained on a limited set of variables, adapted to the context of resource availability in LMICs, may perform satisfactorily compared to models incorporating more extensive laboratory data. These simplified models may find their greatest utility for sepsis screening, for which optimal methods have yet to be determined and are highly context-dependent.

The goal of our exploratory analysis is to encourage further work into the development of tools for the early identification of pediatric sepsis and septic shock in resource-limited settings. The use of ML models in sepsis detection is well-documented. One systematic review in particular examined 28 ML sepsis studies across ICU, hospital ward, and emergency department settings [15]. However, our study aims to address and bring awareness to a gap in the literature. Virtually all studies included in the review were conducted on adult populations in high-income, high-resource settings, using large retrospective electronic health record datasets with rich laboratory and monitoring data, frequently presenting laboratory values as strong predictors of sepsis. In contrast, our study evaluates a pediatric population in a resource-limited setting using simplified feature sets intentionally designed to minimize laboratory requirements. The finding that our non-laboratory models may retain adequate diagnostic performance in contexts where laboratory access is constrained is a finding with direct relevance to the settings largely absent from the existing ML-sepsis evidence base. We, however, recognize that the substantially smaller size (N = 100, vs. 100 s–1000 s in prior studies) [15] and the limitation of our diagnostic assistive tool utilizing PSS components as model inputs indicate that our performance estimates cannot be directly compared to the individual studies within the review and should be treated as preliminary, pending external validation.

Across all evaluated models, logistic regression consistently demonstrated robust and stable performance. Notably, models relying exclusively on clinical variables, including SpO_2_:FiO_2_ ratio, Glasgow Coma Scale (GCS), systolic blood pressure, temperature, heart rate, respiratory rate, and age, performed comparably to models incorporating laboratory data for sepsis classification. This finding suggests that simplified models may retain clinical utility in settings where laboratory testing is unavailable or delayed.

Several variables demonstrated consistent and biologically plausible associations with sepsis and septic shock across models. Notably, lower SpO_2_:FiO_2_ ratio, SBP, and GCS were among the strongest contributors; this finding aligns with existing understanding of sepsis pathophysiology through identification of hypoxemia, cardiovascular dysfunction, and altered mental status, which are early indicators of severe illness and adverse outcomes in pediatric sepsis [25,26,27]. The effect size of these features reinforces the importance of routine, systematic assessment of mental status and complete vital signs in children with suspected infection as part of comprehensive efforts to increase sepsis detection in places with limited diagnostic capacity.

Comparatively, model performance remained high for septic shock, although with a notable discrepancy between AUROC and AUPRC values, highlighting the limitations of AUROC in highly imbalanced datasets and underscoring the importance of precision–recall-based evaluation for rare outcomes. Within this cohort, septic shock occurred in only 23% of the patients, increasing susceptibility to overly optimistic estimates of discrimination. It is critical to note that, while our models’ performance for sepsis and septic shock appears strong, it is highly limited due to our sample size, and no conclusions regarding clinical deployment should be drawn from these results alone. Rather, we encourage building upon our exploratory analysis in developing tools for resource-limited settings with larger sample sizes and external validation.

Notably, our study also demonstrates the adaptability of ML models across contexts by showing the performance of alternate models based on the availability of clinical and laboratory data. Using similar, context-adapted models may also help to promote the use of more accessible organ dysfunction-based sepsis scores, thus contributing to earlier, more sensitive identification of children at risk while addressing the real-world barriers that often impede the global implementation of quality healthcare.

Finally, the expansion of digitalization in health care, including in LMICs, presents new opportunities to incorporate ML models into existing workflows at the point of care through integration into mHealth platforms or electronic health record systems [28]. As technology advances globally, it is critical that clinicians and healthcare workers in LMICs have equitable access to these new tools, adapted to local contextual considerations, and allowing the provision of high-quality evidence-based care for their patients. The real-world implementation of ML-based tools relies on targeted strategies that address the local needs of patients and the healthcare workforce, which can vary significantly by location. For instance, a study on the implementation of a web-based electronic health record system in a tertiary care center in Kenya noted that successful deployment required both strategic planning and broad stakeholder buy-in across clinical, administrative, and IT teams [29]. Important considerations for ML-based tools to be successfully integrated into LMIC health systems include minimizing the number and complexity of input variables, the ability to function on software or applications without internet access, and limited processing requirements to enable real-time decision support [14]. Additionally, successful deployment requires training of personnel of variable comfort with pediatric sepsis to identify children at risk earlier, thereby improving time to treatment and outcomes regardless of the availability of resources in the care setting in which they present [30]. Finally, the healthcare system encompasses more than just healthcare operations; sustainable integration of ML tools necessitates collaboration with policymakers, hospital administrators, and technology developers to ensure these tools align with regulatory, ethical, and cultural standards to protect both providers and patients [31,32].

### Limitations and Future Directions

Several important limitations must be acknowledged when considering this study’s findings. First, we utilized retrospective data from a relatively high-resourced hospital, with a small sample size (N = 100, versus hundreds to thousands often seen in ML models), which limits the generalizability of our findings to other populations. Differences in sepsis epidemiology, healthcare infrastructure, and patient demographics across various regions necessitate follow-up with future external validation studies using much larger datasets. Furthermore, while we aimed to provide variables in our models that would be feasible to obtain for a wide range of hospital settings in LMICs, different environments may still find collecting these variables challenging or impractical, creating difficulties within resource-limited settings. Measuring blood pressure and peripheral oxygen saturation, often omitted in busy, low-resource settings due to human resource or equipment limitations (e.g., pediatric-sized sphygmomanometer cuffs, pulse oximeters), should be a priority in efforts to improve pediatric sepsis care. Recent efforts to scale up pulse oximetry globally, including calls to incorporate oximetry into World Health Organization Integrated Management of Childhood Illness (IMCI) guidelines, are a promising step in the right direction [33].

Another important limitation is the inclusion of GCS and SpO_2_:FiO_2_ ratio as predictor variables, both of which are components of the Phoenix Sepsis Score (PSS) used as our reference standard. This overlap introduces a degree of circularity, as the models may partially “learn” the outcome from predictors embedded within them, likely causing inflated performance metrics such as AUROC, particularly in our models, where feature importance charting placed heavy emphasis on these values. At the same time, the consistent importance of GCS and SpO_2_:FiO_2_ across models reinforces their value as meaningful predictors of sepsis and septic shock, aligned with established pathophysiology and clinical practice. Future work with larger datasets and external validation will be critical in assessing whether performance remains robust. Additionally, GCS may not be universally familiar to all clinicians, and studies evaluating simpler assessments commonly used in pre-hospital and LMIC hospitals, such as AVPU (Awake, Verbal, Pain, and Unresponsive), should be conducted.

Lastly, while ML tools should not replace clinical judgment, they may serve as augmentative aids for healthcare workers to support more systematic and evidence-based screening and decision-making, especially in high-patient-volume contexts where rarer but high-risk conditions like sepsis may be missed. Future work to integrate these models, alternate models for mortality prediction, and models integrating real-time vital signs from wearable devices into novel mHealth tools is planned by this study team.

## 5. Conclusions

Machine learning models developed with a simplified set of clinical variables, contextualized for resource-limited settings, performed adequately in discriminating sepsis from septic shock among children hospitalized with infections in Bangladesh. By minimizing the data requirements, these findings directly address a critical barrier to using organ dysfunction-based sepsis criteria in many LMICs. Our study demonstrates the potential of simplified models as assistive tools in such settings, and we encourage others to build on our exploratory work by incorporating larger populations, conducting external validation, and further refining the features to suit diverse resource settings. As the global health community continues its pursuit of scalable, practical solutions for sepsis management, successful implementation of such models for sepsis screening or referral decisions could enable healthcare workers to recognize sepsis and intervene earlier, thereby reducing morbidity and mortality from sepsis.

## Figures and Tables

**Table 1:** Predictor variables used in machine learning models include baseline values, values taken q12h, and values taken q4h.

Predictors		
2×/Day (q12h)	6×/Day (q4h)	6×/Day (q4h)
Weight	Respiratory Rate	GCS—summed
White Blood Cell	Heart Rate	GCS—Eye Response
Hemoglobin	Temperature	GCS—Motor Response
Platelet Count	Systolic Blood Pressure	GCS—Verbal Response
	Diastolic Blood Pressure	

**Table 2:** Descriptive characteristics of the study population.

Characteristics	Total (N = 100)
Age (months) *	8 [5–18]
Weight (kg) *	6.10 [4.60–9.80]
Sex	
Male	59%
Female	41%
Duration in Hospital (days) *	6 [4–9.75]
In-Hospital Mortality	
Lived	76%
Died	24%
Admission Phoenix Score ≥ 2	37%
Referred From Another Medical Facility	
Yes	21%
No	79%
Past Medical History ^	
None Known	84
Developmental Disability	5
Cerebral Palsy	5
Heart Disease	2
Premature Birth	2
Epilepsy	1
Cleft Palate	1

* Median [IQR = interquartile range]; ^ multiple selections allowed.

**Table 3:** Performance of logistic regression models for sepsis and septic shock classification across feature sets. Models were evaluated using repeated stratified 5-fold cross-validation. Values are reported as point estimates with 95% confidence intervals estimated from 2000 patient-level bootstrap resamples of out-of-fold classifications. Threshold-dependent metrics were calculated using a fixed classification threshold of 0.5.

	Clinical + Lab Sepsis	Non-Lab Sepsis	Clinical + Lab Septic Shock	Non-Lab Septic Shock
Accuracy (95% CI)	0.870 (0.800, 0.930)	0.830 (0.750, 0.900)	0.840 (0.760, 0.910)	0.850 (0.770, 0.920)
F1 Score (95% CI)	0.835 (0.730, 0.914)	0.795 (0.684, 0.879)	0.680 (0.500, 0.808)	0.706 (0.538, 0.833)
Precision (95% CI)	0.846 (0.722, 0.947)	0.767 (0.625, 0.889)	0.630 (0.440, 0.808)	0.643 (0.454, 0.815)
Recall (95% CI)	0.825 (0.698, 0.931)	0.825 (0.692, 0.933)	0.739 (0.542, 0.909)	0.783 (0.593, 0.947)
AUROC (95% CI)	0.945 (0.881, 0.986)	0.945 (0.890, 0.983)	0.870 (0.761, 0.952)	0.878 (0.758, 0.967)
AUPRC (95% CI)	0.941 (0.880, 0.981)	0.942 (0.884, 0.979)	0.793 (0.621, 0.908)	0.822 (0.664, 0.929)
Brier (95% CI)	0.086 (0.049, 0.132)	0.163 (0.149, 0.178)	0.141 (0.119, 0.166)	0.119 (0.080, 0.171)

**Table 4:** Performance of the logistic regression model across Feature Sets 1–3 at three classification thresholds: a conventional threshold (0.50), an inner cross-validation F1-optimal threshold, and a lower screening threshold (0.30). Performance was evaluated using out-of-fold classifications generated from repeated stratified 5-fold cross-validation. Threshold-dependent metrics and confusion matrix components are reported with 95% confidence intervals derived from 2000 patient-level bootstrap resamples. The F1-optimal threshold was determined independently within training folds to avoid information leakage.

Feature Set	Threshold	TP	FP	FN	TN	Sens (95% CI)	Spec (95% CI)	PPV (95% CI)	NPV (95% CI)
Clinical + Lab Sepsis	0.5	33	6	7	54	0.825 (0.698, 0.931)	0.900 (0.814, 0.967)	0.846 (0.722, 0.947)	0.885 (0.797, 0.955)
	0.453	33	6	7	54	0.825 (0.698, 0.931)	0.900 (0.814, 0.967)	0.846 (0.722, 0.947)	0.885 (0.797, 0.955)
	0.3	36	9	4	51	0.900 (0.795, 0.977)	0.850 (0.754, 0.935)	0.800 (0.680, 0.911)	0.927 (0.852, 0.983)
Clinical Sepsis	0.5	33	10	7	50	0.825 (0.692, 0.933)	0.833 (0.732, 0.925)	0.767 (0.625, 0.889)	0.877 (0.783, 0.955)
	0.522	32	4	8	56	0.800 (0.667, 0.917)	0.933 (0.860, 0.985)	0.889 (0.769, 0.975)	0.875 (0.788, 0.951)
	0.3	40	55	0	5	1.000 (1.000, 1.000)	0.083 (0.018, 0.155)	0.421 (0.319, 0.517)	1.000 (1.000, 1.000)
Clinical + Lab Septic Shock	0.5	17	10	6	67	0.739 (0.542, 0.909)	0.870 (0.787, 0.938)	0.630 (0.440, 0.808)	0.918 (0.848, 0.973)
	0.557	16	6	7	71	0.696 (0.481, 0.870)	0.922 (0.853, 0.975)	0.727 (0.524, 0.909)	0.910 (0.843, 0.963)
	0.3	22	41	1	36	0.957 (0.857, 1.000)	0.468 (0.351, 0.580)	0.349 (0.232, 0.468)	0.973 (0.909, 1.000)
Clinical Septic Shock	0.5	18	10	5	67	0.783 (0.593, 0.947)	0.870 (0.789, 0.938)	0.643 (0.454, 0.815)	0.931 (0.866, 0.985)
	0.776	14	4	9	73	0.609 (0.400, 0.800)	0.948 (0.889, 0.988)	0.778 (0.555, 0.947)	0.890 (0.819, 0.951)
	0.3	19	23	4	54	0.826 (0.650, 0.960)	0.701 (0.595, 0.805)	0.452 (0.305, 0.596)	0.931 (0.857, 0.984)

Abbreviations: TP, true positives; FP, false positives; FN, false negatives; TN, true negatives; Sens, sensitivity; Spec, specificity; PPV, positive predictive value; NPV, negative predictive value.

**Table 5:** Standardized logistic regression coefficients and odds ratios for sepsis and septic shock classification across clinical + laboratory and non-laboratory feature sets. Coefficients represent the change in log-odds per one standard deviation increase in each predictor. Odds ratios (ORs) are reported per one standard deviation increase with 95% confidence intervals derived from repeated stratified 5-fold cross-validation. Models were fit using class-weighted logistic regression with standardized predictors. Blank cells indicate features not included in the corresponding feature set.

	Clinical + Lab Sepsis		Non-Lab Sepsis		Clinical + Lab Septic Shock		Non-Lab Septic Shock	
Feature	Coefficient (95% CI)	OR per 1 SD (95% CI)	Coefficient (95% CI)	OR per 1 SD (95% CI)	Coefficient (95% CI)	OR per 1 SD (95% CI)	Coefficient (95% CI)	OR per 1 SD (95% CI)
SpO_2_:FiO_2_ Ratio	−2.783 (−4.270, −1.297)	0.062 (0.014, 0.273)	−0.326 (−0.852, 0.199)	0.722 (0.426, 1.221)	−0.395 (−0.958, 0.168)	0.674 (0.384, 1.183)	−1.458 (−2.357, −0.558)	0.233 (0.095, 0.572)
GCS	−1.596 (−2.874, −0.318)	0.203 (0.056, 0.728)	−0.250 (−0.797, 0.297)	0.779 (0.451, 1.346)	−0.234 (−0.825, 0.358)	0.792 (0.438, 1.430)	−0.402 (−1.167, 0.362)	0.669 (0.311, 1.436)
Systolic Pressure	−1.561 (−3.156, 0.034)	0.210 (0.043, 1.035)	−0.191 (−0.683, 0.301)	0.826 (0.505, 1.352)	−0.512 (−1.099, 0.074)	0.599 (0.333, 1.077)	−2.513 (−4.304, −0.722)	0.081 (0.014, 0.486)
Respiratory Rate	−0.086 (−1.395, 1.222)	0.917 (0.248, 3.393)	0.052 (−0.462, 0.567)	1.054 (0.630, 1.763)	0.145 (−0.405, 0.694)	1.155 (0.667, 2.002)	0.621 (−0.266, 1.509)	1.861 (0.766, 4.523)
Temperature	−0.598 (−1.828, 0.632)	0.550 (0.161, 1.881)	0.011 (−0.506, 0.529)	1.011 (0.603, 1.697)	−0.110 (−0.698, 0.479)	0.896 (0.497, 1.614)	−0.100 (−0.954, 0.755)	0.905 (0.385, 2.127)
Heart Rate	0.689 (−0.314, 1.691)	1.991 (0.731, 5.423)	0.081 (−0.489, 0.651)	1.084 (0.613, 1.917)	0.012 (−0.610, 0.633)	1.012 (0.543, 1.883)	−0.104 (−0.906, 0.699)	0.902 (0.404, 2.012)
Age (months)	0.871 (−0.506, 2.248)	2.389 (0.603, 9.467)	−0.014 (−0.506, 0.477)	0.986 (0.603, 1.612)	0.129 (−0.574, 0.831)	1.137 (0.563, 2.297)	0.895 (0.190, 1.601)	2.448 (1.209, 4.959)
Hemoglobin	−0.508 (−2.120, 1.104)	0.602 (0.120, 3.016)	—	—	−0.013 (−0.672, 0.646)	0.987 (0.511, 1.908)	—	—
Platelet Count	−0.984 (−2.026, 0.058)	0.374 (0.132, 1.059)	—	—	−0.164 (−0.654, 0.326)	0.849 (0.520, 1.385)	—	—
WBC Count	−0.639 (−1.584, 0.306)	0.528 (0.205, 1.358)	—	—	−0.089 (−0.583, 0.404)	0.914 (0.558, 1.498)	—	—

## Data Availability

The dataset presented is openly available on OSF [34]: https://osf.io/bju56/ (accessed on 27 June 2026).

## Supplementary Materials

The following supporting information can be downloaded at: https://www.mdpi.com/article/10.3390/pediatric18040088/s1, Figure S1: Receiver operating characteristic (ROC) and precision–recall (PR) curves for logistic regression models’ classification of sepsis and septic shock across clinical + laboratory and clinical-only feature sets. (A,B) represent ROC and PR curves, respectively, for sepsis classification using the clinical + laboratory feature set. (C,D) represent ROC and PR curves for sepsis classification using only clinical variables. (E,F) show ROC and PR curves for septic shock classification using the clinical+laboratory feature set, while (G,H) display ROC and PR curves for septic shock classification using only clinical variables. Curves were generated using aggregated out-of-fold predicted probabilities obtained from repeated stratified 5-fold cross-validation; Figure S2: Standardized logistic regression feature coefficients for sepsis and septic shock classification across clinical+laboratory and non-laboratory feature sets. (A,B) display feature effects for sepsis classification using the clinical+laboratory and non-laboratory models, respectively, while (C,D) display feature effects for septic shock classification using the corresponding feature sets. Coefficients represent the change in log-odds of the outcome per one standard deviation increase in each predictor. Negative coefficients indicate that lower values of the variable were associated with higher risk, while positive coefficients indicate increased risk with higher values. Feature effects are reported per one standard deviation increase, with 95% confidence intervals derived from repeated stratified 5-fold cross-validation; Table S1: Optimized hyperparameters for each machine learning model across feature sets and outcomes. Hyperparameters were selected using Optuna within a repeated stratified 5-fold cross-validation framework. Only model-specific hyperparameters are reported; parameters not applicable to a given model are omitted for clarity. Feature sets include clinical and laboratory variables (clinical+lab) and clinical variables only (non-lab), evaluated separately for sepsis and septic shock outcomes; Table S2: Model performance for sepsis and septic shock classification across the clinical+laboratory and clinical-only feature sets using repeated stratified 5-fold cross-validation. Values are reported as point estimates with 95% confidence intervals estimated using 2000 patient-level bootstrap resamples of out-of-fold classifications. Threshold-dependent metrics were evaluated at a classification threshold of 0.5; Table S3: Full logistic regression model specifications for sepsis and septic shock across clinical+laboratory (clinical+lab) and clinical-only (non-lab) feature sets. Model equations are expressed in logit form, where *logit(p)* represents the natural logarithm of the odds of the outcome. All predictors were standardized prior to model fitting. Therefore, coefficients represent the change in log-odds of the outcome per one–standard-deviation increase in the corresponding predictor. Intercept terms are reported explicitly for each model.

## Abbreviations

The following abbreviations are used in this manuscript:

AUPRC	Area under the precision–recall curve
AUROC	Area under the receiver operating characteristic curve
KNN	K-nearest neighbor
SVM	Support vector machine
GCS	Glasgow Coma Scale
MAP	Mean arterial pressure
ICU	Intensive care unit
LMIC	Low- and middle-income countries
ML	Machine learning
PSS	Phoenix Sepsis Score
SIRS	Systemic inflammatory response syndrome
mHealth	Mobile health
