# Supplementary material for: Utilizing Machine Learning for Diagnostic Assistance of Pediatric Sepsis and Septic Shock in Resource-Limited Settings
Source: Pediatr Rep. 2026 Jul 3;18(4):88. doi: 10.3390/pediatric18040088 (PMC13398177; doi:10.3390/pediatric18040088)
Supplement: Supplementary file 1 [file pediatrrep-18-00088-s001.zip › pediatrrep-4358533-supplementary.pdf]

Supplementary Materials

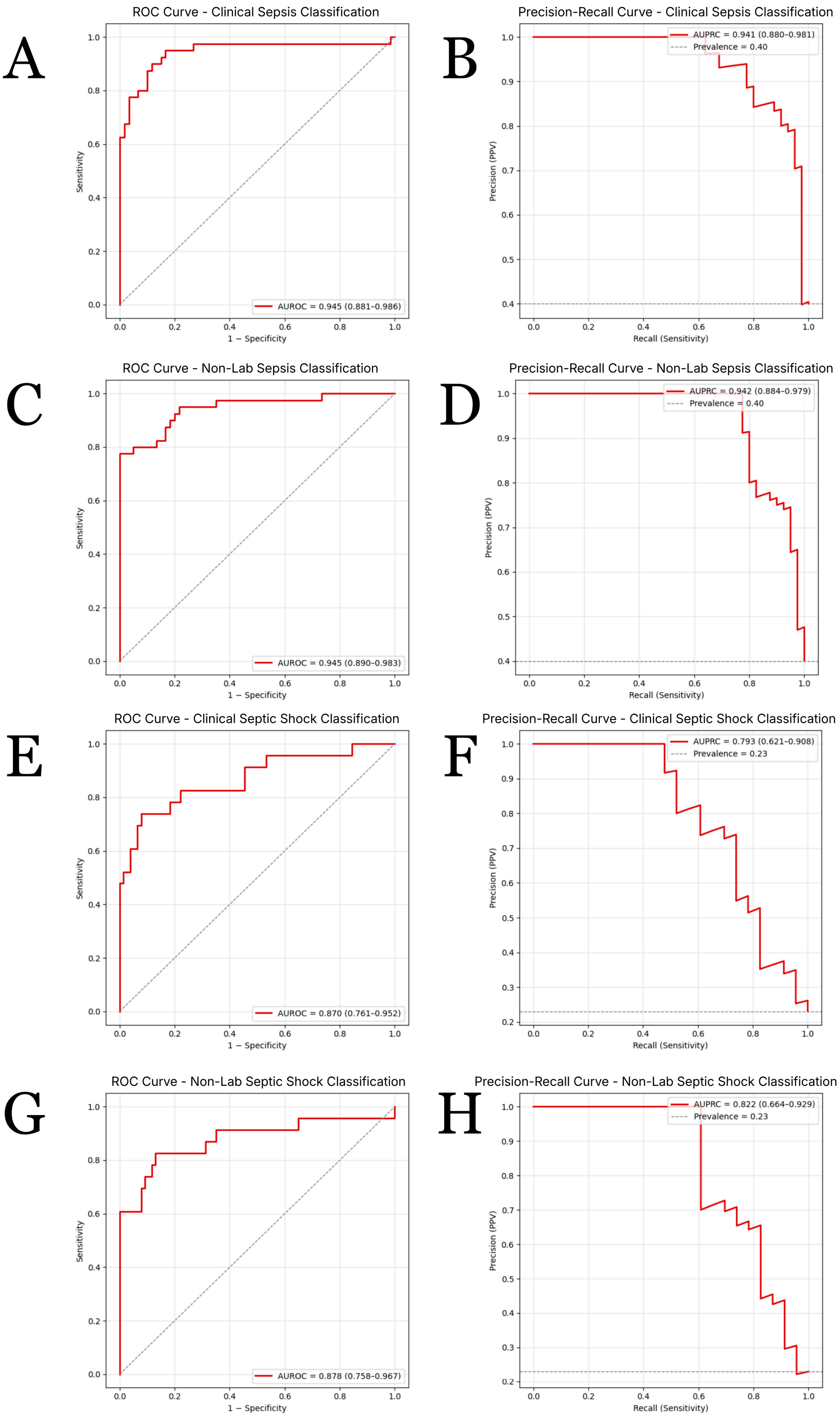

Figure S1. Receiver operating characteristic (ROC) and precision–recall (PR) curves for logistic regression models' classification of sepsis and septic shock across clinical + laboratory and clinical-only feature sets. (**A**,**B**) represent ROC and PR curves, respectively, for sepsis classification using

the clinical + laboratory feature set. (**C**,**D**) represent ROC and PR curves for sepsis classification using only clinical variables. (**E**,**F**) show ROC and PR curves for septic shock classification using the clinical+laboratory feature set, while (**G**,**H**) display ROC and PR curves for septic shock classification using only clinical variables. Curves were generated using aggregated out-of-fold predicted probabilities obtained from repeated stratified 5-fold cross-validation.

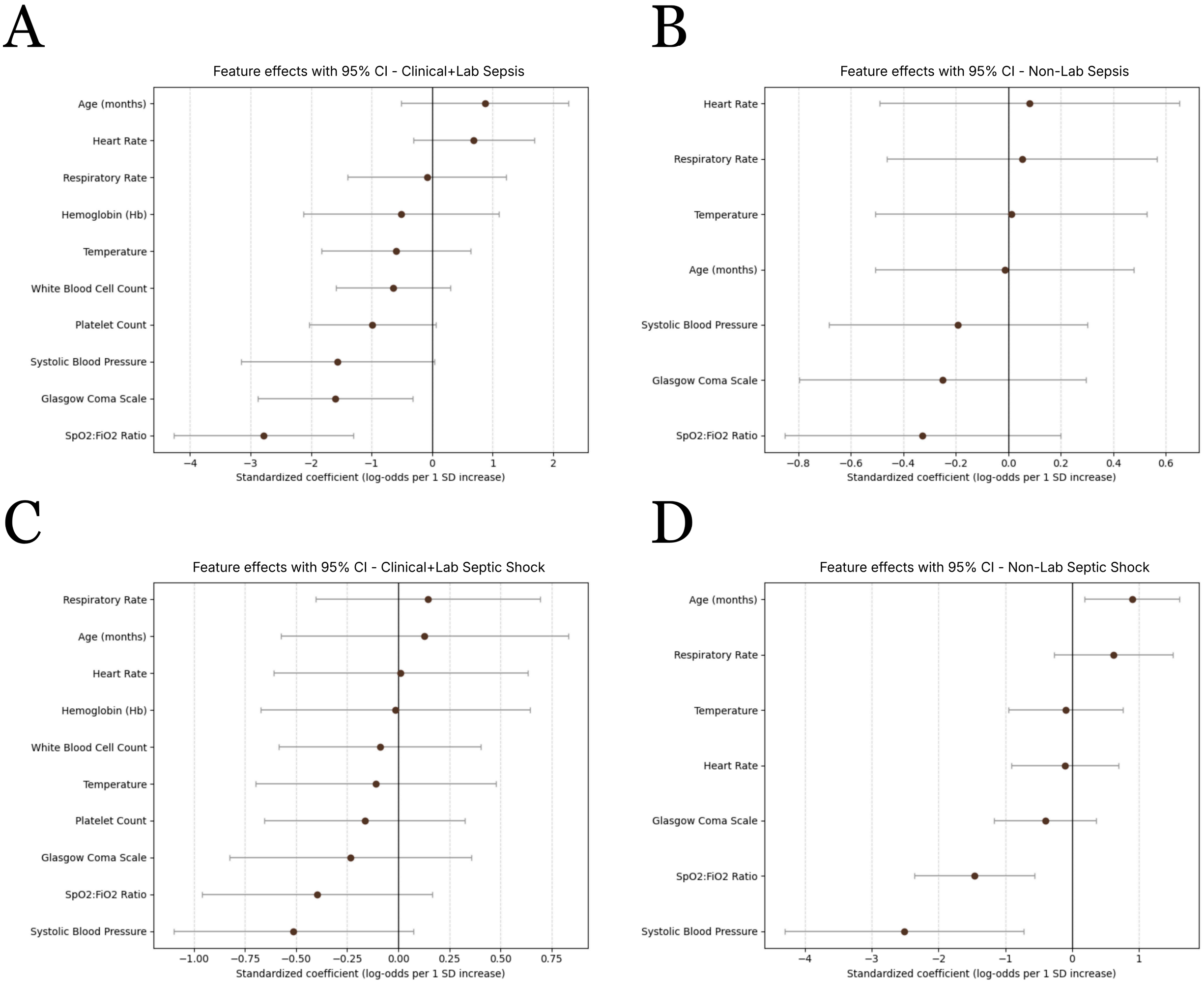

Figure S2. Standardized logistic regression feature coefficients for sepsis and septic shock classification across clinical+laboratory and non-laboratory feature sets. (**A**,**B**) display feature effects for sepsis classification using the clinical+laboratory and non-laboratory models, respectively, while (**C**,**D**) display feature effects for septic shock classification using the corresponding feature sets. Coefficients represent the change in log-odds of the outcome per one standard deviation increase in each predictor. Negative coefficients indicate that lower values of the variable were associated with higher risk, while positive coefficients indicate increased risk with higher values. Feature effects are reported per one standard deviation increase, with 95% confidence intervals derived from repeated stratified 5-fold cross-validation.

Table S1. Optimized hyperparameters for each machine learning model across feature sets and outcomes. Hyperparameters were selected using Optuna within a repeated stratified 5-fold cross-validation framework. Only model-specific hyperparameters are reported; parameters not applicable to a given model are omitted for clarity. Feature sets include clinical and laboratory variables (clinical+lab) and clinical variables only (non-lab), evaluated separately for sepsis and septic shock outcomes.

Feature Set	Model	Hyperparameters
**Clinical+Lab Sepsis**	Decision Tree	max_depth = 8, min_samples_split = 5, min_samples_leaf = 5
	Random Forest	max_depth = 18, min_samples_split = 4, min_samples_leaf = 1, n_estimators = 167
	SVM	C = 0.1419
	Kernel SVM	C = 9.788, gamma = auto
	Naïve Bayes	var_smoothing = 2.283×10^{-8}
	KNN	n_neighbors = 7, weights = distance, p = 1
	Logistic Regression	C = 6.614, solver = lbfgs
**Non-Lab Sepsis**	Decision Tree	max_depth = 7, min_samples_split = 5, min_samples_leaf = 7
	Random Forest	max_depth = 19, min_samples_split = 2, min_samples_leaf = 1, n_estimators = 284
	SVM	C = 0.1073
	Kernel SVM	C = 1.225, gamma = auto
	Naïve Bayes	var_smoothing = 1.522×10^{-8}
	KNN	n_neighbors = 18, weights = distance, p = 1
	Logistic Regression	C = 0.01407, solver = saga
**Clinical+Lab Septic shock**	Decision Tree	max_depth = 3, min_samples_split = 10, min_samples_leaf = 7
	Random Forest	max_depth = 8, min_samples_split = 8, min_samples_leaf = 3, n_estimators = 160
	SVM	C = 0.01055
	Kernel SVM	C = 0.03644, gamma = auto
	Naïve Bayes	var_smoothing = 1.175×10^{-8}
	KNN	n_neighbors = 8, weights = distance, p = 2
	Logistic Regression	C = 0.03661, solver = saga
**Non-Lab Septic shock**	Decision Tree	max_depth = 19, min_samples_split = 10, min_samples_leaf = 1
	Random Forest	max_depth = 3, min_samples_split = 6, min_samples_leaf = 1, n_estimators = 224
	SVM	C = 5.137
	Kernel SVM	C = 0.7318, gamma = scale
	Naïve Bayes	var_smoothing = 7.863×10^{-9}
	KNN	n_neighbors = 15, weights = distance, p = 2
	Logistic Regression	C = 7.828, solver = saga

Table S2. Model performance for sepsis and septic shock classification across the clinical+laboratory and clinical-only feature sets using repeated stratified 5-fold cross-validation. Values are reported as point estimates with 95% confidence intervals estimated using 2000 patient-level bootstrap resamples of out-of-fold classifications. Threshold-dependent metrics were evaluated at a classification threshold of 0.5.

Feature Set	Model	AUROC (95% CI)	PR-AUC (95% CI)	F1 (95% CI)	Precision (95% CI)	Recall (95% CI)	Brier (95% CI)	Accuracy (95% CI)
**Clinical+Lab Sepsis**	Decision Tree	0.880 (0.804, 0.944)	0.848 (0.740, 0.934)	0.744 (0.623, 0.841)	0.763 (0.615, 0.891)	0.725 (0.581, 0.857)	0.124 (0.076, 0.178)	0.800 (0.720, 0.870)
	Random Forest	0.970 (0.936, 0.995)	0.966 (0.925, 0.993)	0.909 (0.829, 0.971)	0.946 (0.857, 1.000)	0.875 (0.758, 0.972)	0.078 (0.055, 0.106)	0.930 (0.870, 0.980)
	SVM	0.960 (0.920, 0.987)	0.947 (0.891, 0.983)	0.833 (0.722, 0.921)	0.938 (0.839, 1.000)	0.750 (0.600, 0.875)	0.092 (0.066, 0.121)	0.880 (0.810, 0.940)
	Kernel SVM	0.946 (0.900, 0.982)	0.920 (0.841, 0.975)	0.831 (0.725, 0.915)	0.865 (0.743, 0.971)	0.800 (0.659, 0.919)	0.101 (0.074, 0.130)	0.870 (0.800, 0.930)
	Naive Bayes	0.920 (0.849, 0.969)	0.912 (0.829, 0.962)	0.815 (0.706, 0.897)	0.805 (0.674, 0.917)	0.825 (0.697, 0.932)	0.119 (0.071, 0.178)	0.850 (0.770, 0.910)
	KNN	0.960 (0.922, 0.987)	0.946 (0.884, 0.984)	0.842 (0.738, 0.921)	0.889 (0.778, 0.976)	0.800 (0.658, 0.914)	0.087 (0.060, 0.119)	0.880 (0.810, 0.940)
	Logistic Regression	0.945 (0.881, 0.986)	0.941 (0.880, 0.981)	0.835 (0.730, 0.914)	0.846 (0.722, 0.947)	0.825 (0.698, 0.931)	0.086 (0.049, 0.132)	0.870 (0.800, 0.930)
**Non-Lab Sepsis**	Decision Tree	0.901 (0.832, 0.958)	0.868 (0.763, 0.945)	0.767 (0.646, 0.865)	0.848 (0.714, 0.966)	0.700 (0.553, 0.839)	0.110 (0.068, 0.157)	0.830 (0.750, 0.900)
	Random Forest	0.968 (0.932, 0.992)	0.963 (0.919, 0.991)	0.907 (0.830, 0.968)	0.971 (0.906, 1.000)	0.850 (0.727, 0.951)	0.074 (0.050, 0.104)	0.930 (0.880, 0.980)
	SVM	0.953 (0.904, 0.987)	0.948 (0.894, 0.983)	0.829 (0.727, 0.907)	0.810 (0.675, 0.925)	0.850 (0.727, 0.950)	0.095 (0.067, 0.128)	0.860 (0.790, 0.920)
	Kernel SVM	0.957 (0.897, 0.994)	0.954 (0.900, 0.992)	0.875 (0.782, 0.944)	0.875 (0.762, 0.974)	0.875 (0.756, 0.971)	0.078 (0.052, 0.110)	0.900 (0.840, 0.950)
	Naive Bayes	0.926 (0.856, 0.975)	0.925 (0.853, 0.969)	0.847 (0.753, 0.922)	0.800 (0.673, 0.911)	0.900 (0.791, 0.976)	0.107 (0.060, 0.165)	0.870 (0.800, 0.930)
	KNN	0.949 (0.904, 0.982)	0.936 (0.877, 0.979)	0.853 (0.750, 0.930)	0.914 (0.806, 1.000)	0.800 (0.659, 0.919)	0.098 (0.068, 0.134)	0.890 (0.820, 0.950)
	Logistic Regression	0.945 (0.890, 0.983)	0.942 (0.884, 0.979)	0.795 (0.684, 0.879)	0.767 (0.625, 0.889)	0.825 (0.692, 0.933)	0.163 (0.149, 0.178)	0.830 (0.750, 0.900)
**Clinical+Lab Septic shock**	Decision Tree	0.768 (0.642, 0.870)	0.551 (0.350, 0.749)	0.490 (0.300, 0.646)	0.462 (0.261, 0.650)	0.522 (0.316, 0.727)	0.179 (0.126, 0.239)	0.750 (0.660, 0.830)
	Random Forest	0.851 (0.749, 0.932)	0.728 (0.546, 0.861)	0.619 (0.421, 0.769)	0.684 (0.461, 0.882)	0.565 (0.364, 0.760)	0.120 (0.089, 0.155)	0.840 (0.760, 0.910)
	SVM	0.878 (0.775, 0.954)	0.793 (0.624, 0.910)	0.595 (0.359, 0.765)	0.786 (0.538, 1.000)	0.478 (0.250, 0.684)	0.098 (0.064, 0.137)	0.850 (0.780, 0.920)
	Kernel SVM	0.881 (0.791, 0.947)	0.755 (0.573, 0.884)	0.556 (0.323, 0.724)	0.769 (0.500, 1.000)	0.435 (0.222, 0.636)	0.111 (0.078, 0.147)	0.840 (0.760, 0.910)
	Naive Bayes	0.826 (0.683, 0.937)	0.773 (0.592, 0.895)	0.681 (0.486, 0.816)	0.667 (0.455, 0.846)	0.696 (0.481, 0.870)	0.128 (0.073, 0.192)	0.850 (0.770, 0.910)
	KNN	0.868 (0.766, 0.946)	0.763 (0.593, 0.886)	0.414 (0.160, 0.621)	1.000 (1.000, 1.000)	0.261 (0.087, 0.450)	0.120 (0.076, 0.170)	0.830 (0.750, 0.900)
	Logistic Regression	0.870 (0.761, 0.952)	0.793 (0.621, 0.908)	0.680 (0.500, 0.808)	0.630 (0.440, 0.808)	0.739 (0.542, 0.909)	0.141 (0.119, 0.166)	0.840 (0.760, 0.910)
**Non-Lab Septic Shock**	Decision Tree	0.808 (0.692, 0.902)	0.629 (0.422, 0.800)	0.583 (0.390, 0.731)	0.560 (0.364, 0.750)	0.609 (0.400, 0.800)	0.150 (0.096, 0.215)	0.800 (0.720, 0.870)
	Random Forest	0.866 (0.763, 0.947)	0.783 (0.613, 0.897)	0.700 (0.500, 0.837)	0.824 (0.615, 1.000)	0.609 (0.391, 0.800)	0.114 (0.086, 0.145)	0.880 (0.810, 0.940)
	SVM	0.875 (0.760, 0.958)	0.803 (0.630, 0.916)	0.606 (0.370, 0.788)	1.000 (1.000, 1.000)	0.435 (0.227, 0.650)	0.102 (0.068, 0.142)	0.870 (0.800, 0.930)
	Kernel SVM	0.904 (0.824, 0.963)	0.802 (0.639, 0.914)	0.700 (0.500, 0.839)	0.824 (0.611, 1.000)	0.609 (0.400, 0.800)	0.098 (0.067, 0.133)	0.880 (0.810, 0.940)
	Naive Bayes	0.842 (0.701, 0.947)	0.796 (0.629, 0.910)	0.711 (0.516, 0.842)	0.727 (0.519, 0.895)	0.696 (0.481, 0.870)	0.113 (0.064, 0.175)	0.870 (0.800, 0.930)
	KNN	0.887 (0.799, 0.952)	0.796 (0.632, 0.910)	0.414 (0.160, 0.615)	1.000 (1.000, 1.000)	0.261 (0.087, 0.444)	0.128 (0.083, 0.176)	0.830 (0.760, 0.900)
	Logistic Regression	0.878 (0.758, 0.967)	0.822 (0.664, 0.929)	0.706 (0.538, 0.833)	0.643 (0.454, 0.815)	0.783 (0.593, 0.947)	0.119 (0.080, 0.171)	0.850 (0.770, 0.920)

Table S3. Full logistic regression model specifications for sepsis and septic shock across clinical+laboratory (clinical+lab) and clinical-only (non-lab) feature sets. Model equations are expressed in logit form, where *logit(p)* represents the natural logarithm of the odds of the outcome. All predictors were standardized prior to model fitting. Therefore, coefficients represent the change in log-odds of the outcome per one–standard-deviation increase in the corresponding predictor. Intercept terms are reported explicitly for each model.

Feature Set	Outcome	Model Specification
**Clinical+Lab**	Sepsis	logit(p) = −0.335 + (−2.783 × SpO2:FiO2 Ratio) + (−0.639 × White Blood Cell Count) + (−0.598 × Temperature) + (−0.984 × Platelet Count) + (0.689 × Heart Rate) + (−0.508 × Hemoglobin (Hb)) + (−0.086 × Respiratory Rate) + (−1.561 × Systolic Blood Pressure) + (0.871 × Age (months)) + (−1.596 × Glasgow Coma Scale)
**Non-Lab**	Sepsis	logit(p) = −0.092 + (−0.326 × SpO2:FiO2 Ratio) + (0.011 × Temperature) + (0.081 × Heart Rate) + (0.052 × Respiratory Rate) + (−0.191 × Systolic Blood Pressure) + (−0.014 × Age (months)) + (−0.250 × Glasgow Coma Scale)
**Clinical+Lab**	Septic Shock	logit(p) = −0.411 + (−0.395 × SpO2:FiO2 Ratio) + (−0.089 × White Blood Cell Count) + (−0.110 × Temperature) + (−0.164 × Platelet Count) + (0.012 × Heart Rate) + (−0.013 × Hemoglobin (Hb)) + (0.145 × Respiratory Rate) + (−0.512 × Systolic Blood Pressure) + (0.129 × Age (months)) + (−0.234 × Glasgow Coma Scale)
**Non-Lab**	Septic Shock	logit(p) = −0.884 + (−1.458 × SpO2:FiO2 Ratio) + (−0.100 × Temperature) + (−0.104 × Heart Rate) + (0.621 × Respiratory Rate) + (−2.513 × Systolic Blood Pressure) + (0.895 × Age (months)) + (−0.402 × Glasgow Coma Scale)
